# FTH1- and SAT1-Induced Astrocytic Ferroptosis Is Involved in Alzheimer’s Disease: Evidence from Single-Cell Transcriptomic Analysis

**DOI:** 10.3390/ph15101177

**Published:** 2022-09-22

**Authors:** Yini Dang, Qing He, Siyu Yang, Huaiqing Sun, Yin Liu, Wanting Li, Yi Tang, Yu Zheng, Ting Wu

**Affiliations:** 1 Department of Gastroenterology, the First Affiliated Hospital of Nanjing Medical University, Nanjing 210029, China; 2 Division of Gastroenterological Rehabilitation, Department of Gastroenterology, the First Affiliated Hospital of Nanjing Medical University, Nanjing 210029, China; 3 Department of Neurology, the Affiliated Hospital of China University of Mining and Technology, Xuzhou 221116, China; 4 Division of Brain Rehabilitation, Department of Neurology, the First Affiliated Hospital of Nanjing Medical University, No.300 Guangzhou Road, Nanjing 210029, China; 5 Department of Neurology, the First Affiliated Hospital of Nanjing Medical University, No.300 Guangzhou Road, Nanjing 210029, China; 6 Department of Rehabilitation Medicine, the First Affiliated Hospital of Nanjing Medical University, No.300 Guangzhou Road, Nanjing 210029, China

**Keywords:** Alzheimer’s disease, single-cell RNA sequencing, astrocytes, ferroptosis

## Abstract

Objectives: Despite significant advances in neuroscience, the mechanisms of AD are not fully understood. Single-cell RNA sequencing (scRNA-seq) techniques provide potential solutions to analyze cellular composition of complex brain tissue and explore cellular and molecular biological mechanisms of AD. Methods: We investigated cellular heterogeneity in AD via utilization of bioinformatic analysis of scRNA-seq in AD patients and healthy controls from the Gene Expression Omnibus (GEO) database. The “GOplot” package was applied to explore possible biological processes in oligodendrocytes, astrocytes, and oligodendrocyte progenitor cells (OPCs). Expression patterns and biological functions of differentially expressed genes (DEGs) from scRNA-seq data were validated in RNA sequencing data. DEGs in astrocytes interacted with ferroptosis-related genes in FerrDb. CCK-8 and EdU assays were performed to measure cell proliferation ability. ROS, Fe^2+^ level, mitochondrial membrane potentials, iron concentrations, and total iron binding capacity (TIBC) in serum were evaluated. Y-maze and elevated maze were used to measure anxiety-like behavior. Autonomous and exploration behaviors or learning and memory ability in mice were analyzed using open field test and novel object recognition test. Results: Multiple clusters were identified, including oligodendrocytes, astrocytes, OPCs, neurons, microglia, doublets, and endothelial cells. Astrocytes were significantly decreased in AD, while oligodendrocytes and OPCs increased. Cell-to-cell ligand–receptor interaction analysis revealed that astrocytes, neurons, and OPCs mainly established contacts with other cells via the NRG3–ERBB4 ligand–receptor pair. GO and KEGG analyses found that astrocytes were enriched in the ferroptosis pathway. FTH1 and SAT1 in astrocytes were identified as hub mRNAs associated with ferroptosis. Serum iron concentration of 5xFAD mice was higher than that of WT, and emotional and cognitive function were significantly impaired as compared to WT. Serum iron concentration was negatively correlated with number of astrocytes and percentage of time spent entering the novelty arm in the Y-maze test, while it was positively correlated with percentage of time spent in the central area. Meanwhile, number of astrocytes was negatively correlated with percentage of time spent in the central area, while it was positively correlated with percentage of time spent entering the novelty arm. Conclusions: Through scRNA-seq analysis, we found that ferroptosis was activated in astrocytes and may contribute to the pathophysiological process in the entorhinal cortex. FTH1 and SAT1 were identified to impact astrocyte ferroptosis. Emotional and cognitive impairment in AD was associated with astrocyte ferroptosis. Our findings provide clues to reveal the pathophysiological processes following AD at the cellular level and highlight potential drug targets for the treatment of AD.

## 1. Introduction

Alzheimer’s disease (AD), the most common form of progressive dementia, is an incurable neurodegenerative disease [1]. The global prevalence is over 50 million, and no effective intervention exists. AD is among the leading contributors to medical and financial burden [2]. In this context, advanced therapy for AD remains a warranted need.

Exploring the molecular mechanisms underlying AD is essential for the development of innovative therapies. Over the past few decades, extensive efforts have revealed that AD is featured with deposition of amyloid-β (Aβ) [3,4,5] and tau neurofibrillary tangles (NFTs) [6]. Neurodegenerative changes in AD begin in the entorhinal cortex and hippocampus, then propagate in a stereotypical manner [7]. With support of genomic, transcriptomic, and epigenomic analyses, knowledge regarding the transcriptional network of AD has been expanded. For instance, a pan-cortical brain region genomic analysis of AD revealed that cytoskeleton, axon guidance, and nervous system development pathways were associated with AD pathology [8]. On the other hand, bulk tissue analyses represented changes in cell composition and obscured precise cellular changes [9]. Molecular and cellular heterogeneity may also play a role in AD pathophysiology [10,11]. Therefore, in-depth investigation into cellular heterogeneity would be helpful in the exploration of interventional targets in AD.

Recently, rapid progress in next-generation sequencing (NGS) technologies has delivered insights into complex biological processes [12]. Genomics, transcriptomics, and epigenomics are now increasingly applied in the characterization of individual cells. Additionally, single-cell RNA sequencing (scRNA-seq) offers possibilities to dissect cellular heterogeneity. It is capable to uncover regulatory relationships between genes and track trajectories of distinct cell lineages in development [13]. Application of scRNA-seq in AD has provided a molecular atlas of the brain with unparalleled resolution, explored molecular vulnerability of specific neuronal subpopulations, and identified candidate target molecules for therapeutic intervention [14]. Keren-Shaul and colleagues identified a subcluster of microglia-disease-associated microglia (DAM) in a mouse model of AD by scRNA-seq [15]. DAMs were featured with microglial markers, including Iba1, Cst3, and Hexb, and were demonstrated to be central to lysosome, phagocyte, and lipid metabolism pathways [16]. Additionally, Mathys and colleagues performed single-nucleus RNA sequencing to explore gene deviation in the prefrontal cortex region of patients with multiple AD pathologies [17]. It demonstrated that major cell types in the prefrontal cortex were affected at a transcriptional level. In excitatory and inhibitory neurons, most differentially expressed genes (DEGs) were downregulated, while DEGs in astrocytes, microglia, and oligodendrocytes showed an upregulation trend [17]. Although these findings partially provided new insights into treatment strategies, understanding of the whole picture and specific cellular interactions is warranted with advanced NGS techniques.

Here, we investigated cellular heterogeneity in AD via bioinformatic analysis of scRNA-seq of the entorhinal cortex region in AD patients and healthy controls from the Gene Expression Omnibus (GEO) database. Intercellular communication analysis was performed to explore potential interactions between cells in the entorhinal cortex. We demonstrated that cell-type-specific changes in oligodendrocytes, astrocytes, and oligodendrocyte precursor cells (OPCs) were relevant with dysregulation of oxidative phosphorylation, ferroptosis, and synaptic signaling. Afterwards, we interacted the DEGs in astrocytes with genes related to ferroptosis in open-source datasets. We finally verified the regulation of ferroptosis in astrocytes by selected hub mRNAs and found that emotional and cognitive impairment in AD was associated with astrocyte ferroptosis. Collectively, our scRNA-seq analysis of the entorhinal cortex region provided novel insights into the cellular mechanisms underlying AD. This provides an opportunity for the identification of novel diagnostic molecular markers, as well as novel therapeutic targets, on the basis of which more effective management strategies for AD are being developed.

## 2. Results

**Figure 1:**
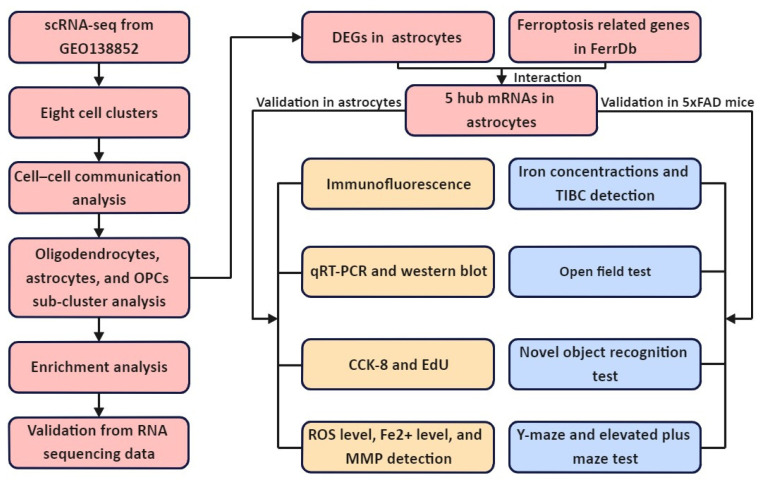
Study flowchart. scRNA-seq: single-cell RNA sequencing. OPCs: oligodendrocyte progenitor cells. DEGs: differentially expressed genes. qRT-PCR: quantitative real-time reverse transcription-polymerase chain reaction. CCK-8: cell counting kit-8. EdU: 5-ethynyl-20-deoxyuridine. MMP: mitochondrial membrane potential. TIBC: total iron binding capacity.

The detailed procedure of scRNA-seq analysis and validation of newly discovered hub mRNAs on ferroptosis in astrocytes are demonstrated in Figure 1.

### 2.1. scRNA Profiling of Human Entorhinal Cortex in AD

**Figure 2:**
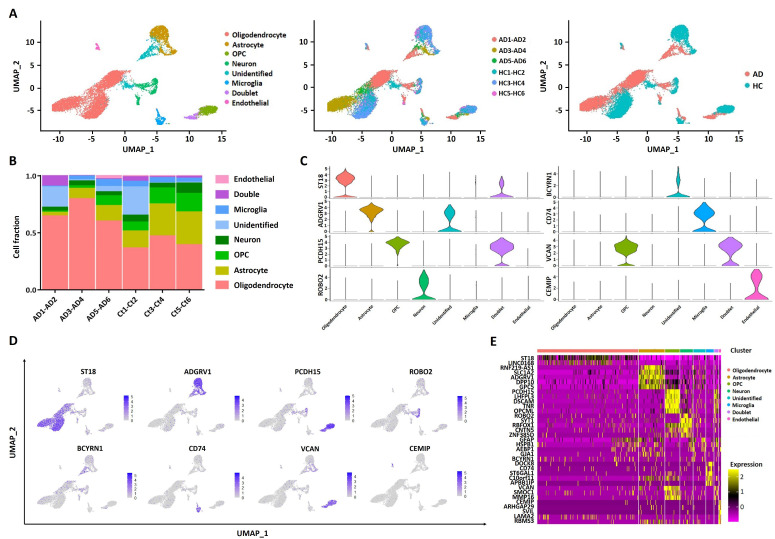
Single−cell profiling of entorhinal cortex from AD patients and healthy controls. (**A**) UMAP plots of identified cells show the components in entorhinal cortex region, color−coded by the major cell lineage (**left**), corresponding patient (**middle**), and sample type (**right**). (**B**) Fraction of major cell lineages. (**C**) Violin plots of hub genes across eight clusters. (**D**) Normalized expression of hub genes for each cell type. (**E**) Heatmap of top five DEGs in each cluster. HC: healthy controls. AD: Alzheimer’s disease.

The scRNA-seq dataset (GSE138852) of the entorhinal cortex region in AD patients and healthy controls from the GEO database was collected and analyzed. After quality filtering of gene expression normalization for read depth and mitochondrial read count, 6564 out of 13,096 cells from AD patients and 6532 from healthy controls were retained. We applied principal component analysis and a graph-based clustering approach to categorize individual cells into eight clusters, including oligodendrocytes (featured with ST18 and CTNNA3), astrocytes (featured with SLC1A2 and ADGRV1), oligodendrocyte progenitor cells (OPCs, featured with PCDH15 and LHFPL3), neurons (featured with ROBO2 and SYT1), microglia (featured with DOCK8 and CD74), doublets (featured with PCDH15 and VCAN), endothelial cells (featured with CEMIP and ARHGAP29), and unidentified cells (featured with BCYRN1 and GJA1) (Figure 2A). Among these cells, oligodendrocytes, astrocytes, and OPCs were mostly enriched in the entorhinal cortex (Figure 2B, Table S2 in Supplementary File SI). A demonstration of the hub genes central to the cell types is shown in a violin plot (Figure 2C) and UMAP plot (Figure 2D). The top five DEGs in each cluster are shown in Figure 2E, and information regarding the hub genes in each cluster is listed in Supplementary File SII.

### 2.2. Identification of Significant Intercellular Communication in Entorhinal Cortex

**Figure 3:**
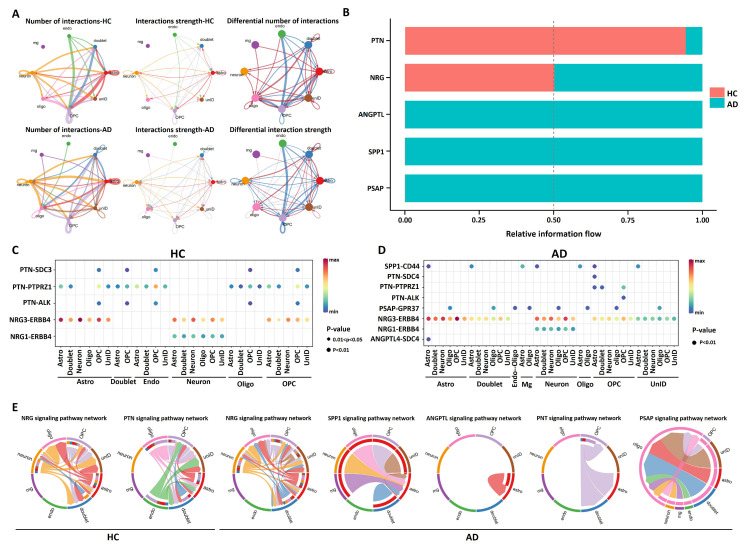
Intercellular ligand–receptor prediction. (**A**) Number and strength of interactions between AD and controls. (**B**) Histogram of proportion of PTN, NRG, ANGPTL, SPP1, and PSAP pathways between AD and HC. (**C**) Dot plot of interactions between cell types in controls. (**D**) Dot plot of interactions between cell types in AD. (**E**) Chord diagrams of intercellular communication between signaling pathways. HC: healthy controls. AD: Alzheimer’s disease. Astro: astrocytes. UnID: unidentified cells. Oligo: oligodendrocytes. Endo: endothelial cells. Mg: microglia.

To explore interactions and signaling networks across different cell clusters in AD versus control, the expression of various ligand–receptor pairs was measured. CellChat detected a number of significant ligand–receptor pairs in eight cell clusters, among which astrocytes, OPCs, and neurons appeared to show stronger intercellular communication across cell clusters in AD (Figure 3A). Afterwards, ligand–receptor pairs were further categorized into five signaling pathways, including PTN, NRG, ANGPTL, SPP1, and PSAP signaling pathways. We found that the PTN signaling pathway was dominant in controls, while the ANGPTL, SPP1, and PSAP signaling pathways were significantly enriched in AD (Figure 3B,C, Supplementary File SIII–SIV). As shown in Figure 3D, astrocytes, neurons, and OPCs established contacts with other cells via the NRG3–ERBB4 ligand–receptor pair.

### 2.3. Heterogeneity of Oligodendrocytes, Astrocytes, and OPCs in AD

**Figure 4:**
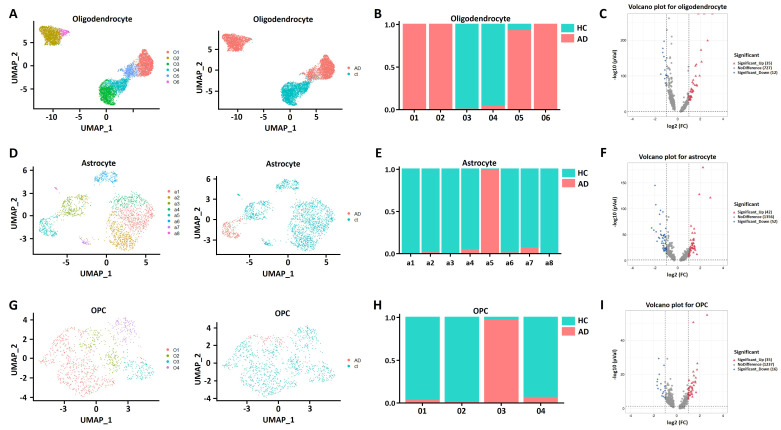
Overview of cell subsets. (**A**) UMAP visualization of subclustered oligodendrocytes colored by major cell lineage (**left**) and sample type (right). (**B**) Fraction of subclustered oligodendrocytes. (**C**) Volcano plot of DEGs in oligodendrocytes. (**D**) UMAP visualization of subclustered astrocytes colored by major cell lineage (**left**) and sample type (**right**). (**E**) Fraction of sub-clustered astrocytes. (**F**) Volcano plot of DEGs in astrocytes. (**G**) UMAP visualization of subclustered OPCs colored the major cell lineage (**left**) and sample type (right). (**H**) Fraction of subclustered OPCs. (**I**) Volcano plot of DEGs in OPCs. FC: fold change. pVal: *p* value.

**Figure 5:**
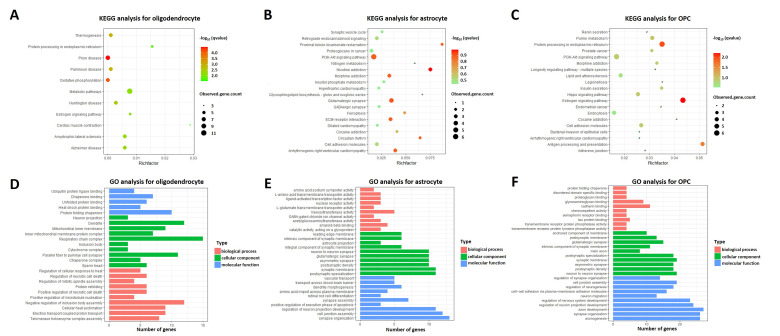
Enrichment analysis of DEGs in oligodendrocytes, astrocytes, and OPCs. (**A**) KEGG analysis of DEGs in oligodendrocytes. (**B**) KEGG analysis of DEGs in astrocytes. (**C**) KEGG analysis of DEGs in OPCs. (**D**) GO analysis of DEGs in oligodendrocytes. (**E**) GO analysis of DEGs in astrocytes. (**F**) GO analysis of DEGs in OPCs. GO: Gene Ontology. KEGG: Kyoto Encyclopedia of Genes and Genomes.

According to our scRNA-seq analysis, significant differences in number of oligodendrocytes, astrocytes, and OPCs were detected between AD patients and healthy controls. Hence, we further performed heterogeneity analysis. Six different subclusters were reclustered in oligodendrocytes (Figure 4A,B). Furthermore, 35 mRNAs (e.g., HSPA1A, FKBP5, and HSPB1) were significantly upregulated, and 12 mRNAs (e.g., CTNNA2, PDE1A, and ZFYVE16) were downregulated in oligodendrocytes. Volcano plots of DEGs are shown in Figure 4C. Based on our GO and KEGG analyses, oligodendrocytes were markedly enriched in prion disease, oxidative phosphorylation, and metabolic pathways (Figure 5A,D). Meanwhile, a total of 2171 astrocytes and eight subclusters, derived from AD or healthy controls, were detected (Figure 4D,E). Compared with controls, 42 mRNAs (e.g., LINGO1, NEAT1, and GFAP) were significantly upregulated, while 52 mRNAs (e.g., LSAMP-AS1, SLC6A1-AS1, and NRXN1) downregulated in AD (Figure 4F). As shown in Figure 5B,E, the DEGs of astrocytes were associated with the PI3K-AKT signaling pathway, astrocyte projection, and ferroptosis. In addition, four subclusters were identified in 1078 OPCs. UMAP plots of OPCs subclusters in AD and controls are shown in Figure 4G,H. Further, 35 mRNAs (e.g., MT-ND4, MT-CYB, and XIST) were significantly upregulated, while 16 mRNAs (e.g., CNTN5, GPC5, and RBFOX1) were downregulated in OPCs (Figure 4I). The results of GO and KEGG enrichment analyses on the DEGs of OPCs indicated that OPCs were central to neuron-to-neuron synapse, especially for protein processing in the endoplasmic reticulum and estrogen signaling pathway (Figure 5C,F).

### 2.4. DEGs from RNA Sequencing Data

**Figure 6:**
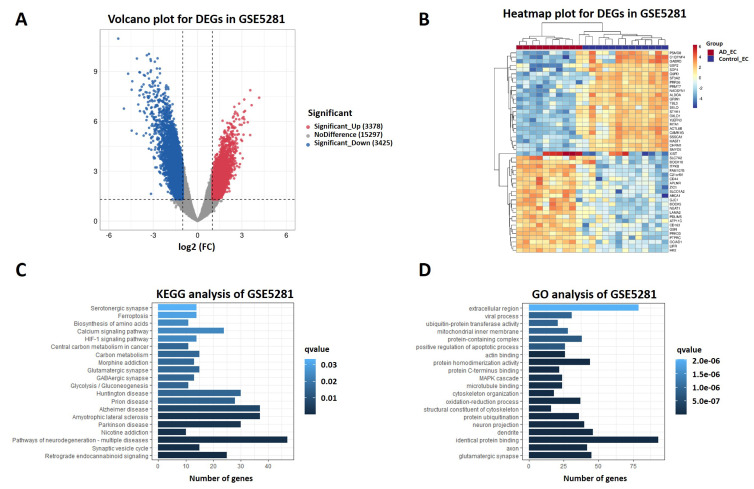
DEGs of AD from GSE5281 dataset. (**A**) Volcano plot of DEGs in GSE5281. Upregulated genes were colored in red and downregulated genes in blue. (**B**) Heatmap of top 25 upregulated and top 25 downregulated DEGs in GSE5281. (**C**) GO analysis of DEGs in GSE5281. (**D**) KEGG analysis of DEGs in GSE5281. GO: Gene Ontology. KEGG: Kyoto Encyclopedia of Genes and Genomes. DEGs: differentially expressed genes. FC: fold change. EC: entorhinal cortex.

To validate the expression features of the entorhinal cortex region in AD, RNA sequencing dataset GSE5281, which included 10 AD patients and 13 controls, was analyzed with the “limma” package to identify DEGs affecting AD−related pathways or biological processes. DEGs were selected with a *p* value < 0.05 and an absolute logFC > 1. 3378 upregulated and 3425 downregulated DEGs were identified. Accordingly, expression levels of HSPA1A, LINGO1, and XIST were upregulated, while PDE1A, NRXN1, and RBFOX1 were downregulated. Volcano and heatmap plots of DEGs are shown in Figure 6A,B. Similar to the expression features of scRNA−Seq, DEGs in GSE5281 were also central to ferroptosis and neuron projection (Figure 6C,D).

### 2.5. In Vitro Validation of Astrocyte Ferroptosis

**Figure 7:**
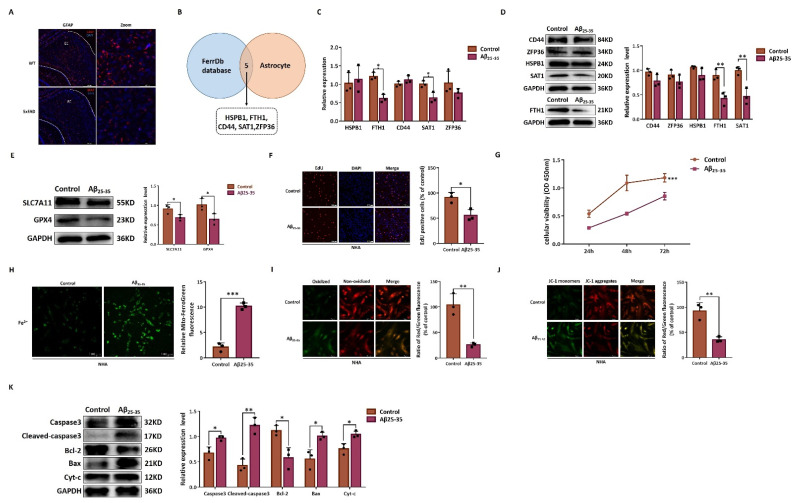
In vitro validation of astrocyte ferroptosis. (**A**) Representative immunofluorescence images of GFAP protein expression in entorhinal cortex region. GFAP-positive astrocytes (red) and DAPI-stained nuclei (blue). (**B**) Venn diagram to interact DEGs in astrocytes with genes related to ferroptosis in FerrDb. (**C**) Expression of HSPB1, FTH1, CD44, SAT1, and ZFP36 by qRT-PCR. (**D**) Expression of HSPB1, FTH1, CD44, SAT1, and ZFP36 by Western blot. (**E**) Expression of SLC7A11 and GPX4 by Western blot. (**F**) Cell proliferation by EdU assay. (**G**) Cell viability by CCK-8 assay. (**H**) Representative image Mito-FerroGreen staining of Fe^2+^. (**I**) Cellular lipid ROS level by C11-BODIPY581/591 dye. Green fluorescence represents oxidized form; red fluorescence represents non-oxidized form. (**J**) Mitochondrial membrane potential by JC-1. (**K**) Expression of Caspase-3, Cleaved-caspase3, Bcl-2, Bax, and Cyt-c by Western blot. Red, JC-1 aggregates; green, JC-1 monomer. WT: wild type. FAD: familial Alzheimer’s disease. EC: entorhinal cortex. EdU: 5-ethynyl-20-deoxyuridine. DAPI: 4′,6-diamidine-2′-phenylindole dihydrochloride. NHA: normal human astrocytic cells. * *p* < 0.05; ** *p* < 0.01; *** *p* < 0.001.

Immunofluorescence in the entorhinal cortex observed a decreased number of GFAP-positive astrocytes in 5xFAD mice (Figure 7A). We then interacted DEGs in astrocytes with genes related to ferroptosis in FerrDb. HSPB1, FTH1, CD44, SAT1, and ZFP36 were finally identified to be significantly associated with ferroptosis (Figure 7B). Gene and protein expression were compared between the Aβ_25–35_ and control group by qRT-PCR and Western blot. After Aβ_25–35_ treatment, FTH1 and SAT1 were significant downregulated (Figure 7C,D). GPX4 and SLC7A11 showed a decreased trend in protein level in the Aβ_25–35_ group as compared to that in the control group (Figure 7E). Cell viability and EdU-positive cells were significantly decreased due to Aβ_25–35_ injury (Figure 7F,G). Fe^2+^ and ROS level were significantly increased in the Aβ_25–35_ group, while mitochondrial membrane potential was markedly decreased (Figure 7H–J). Intracellular signaling involved downstream collapse in mitochondrial membrane potential and was characterized with increased protein level of Caspase3, Cleaved-caspase3, Bax, and Cyt-c and decreased Bcl-2 in the Aβ25–35 group as compared with the control group (Figure 7K).

### 2.6. 5xFAD Mice Developed Emotional and Cognitive Impairment

**Figure 8:**
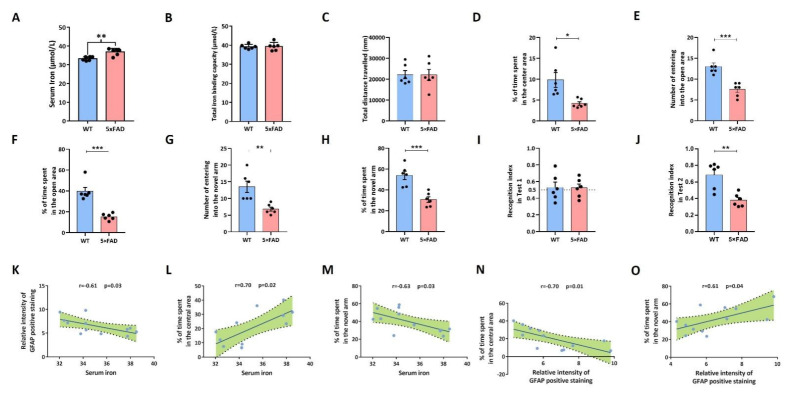
In vivo emotional and cognitive impairment validation. (**A**) Serum iron concentration. (**B**) Total iron binding capacity. (**C**) Total movement distance. (**D**) Percentage of time spent in the central area. (**E**) Number of mice entering open arm in elevated cross test. (**F**) Percentage of time spent entering open arm in elevated cross test. (**G**) Number of mice entering novelty arm in Y-maze test. (**H**) Percentage of time spent entering novelty arm in Y-maze test. (**I**) Percentage of sniffing exercise time between two identical objects in the first stage of novel object recognition. (**J**) Percentage of sniffing exercise time between mice in the second stage of novel object recognition. Black circle represents number of mice. (**K**) Correlation between serum iron concentration and relative intensity of GFAP positive staining, r = −0.61, *p* = 0.03. (**L**) Correlation between serum iron concentration and percentage of time spent in the central area, r = 0.70, *p* = 0.02. (**M**) Correlation between serum iron concentration and percentage of time spent entering novelty arm in Y-maze test, r = −0.63, *p* = 0.03. (**N**) Correlation between relative intensity of GFAP positive staining and percentage of time spent in the central area, r = −0.70, *p* = 0.01. (**O**) Correlation between relative intensity of GFAP positive staining and percentage of time spent entering novelty arm in Y-maze test, r = 0.61, *p* = 0.04. WT: wild type. FAD: familial Alzheimer’s disease. * *p* < 0.05; ** *p* < 0.01; *** *p* < 0.001.

To further demonstrate the role of ferroptosis in AD, we elucidated the disturbance of iron metabolism and conducted behavioral tests in 5xFAD mice (5xFAD mice recapitulate key features of Alzheimer’s amyloid pathology and serve as a model for Aβ-42-induced neurodegeneration and amyloid plaque formation in neurons). We measured the serum iron concentration and total iron binding capacity. Compared with WT mice, the iron level in the serum was significant increased (Figure 8A). The total iron binding capacity values were comparable to those of their littermate controls (Figure 8B). Afterwards, an open field test and elevated maze test were performed to explore anxious-like behavior. For the open field test, there was a significant difference in percentage of time spent in the central area, while no difference was detected in the total movement distance between 5xFAD and WT mice (Figure 8C,D). In addition, the results of the elevated maze test revealed that the number of entries and percentage of time spent in the open arm of the elevated cross were significantly decreased in 5xFAD mice compared with WT mice (Figure 8E,F). Subsequently, the Y-maze test and novel object recognition test were conducted to assess cognitive function. In the Y-maze test, time spent entering the NA and percentage of time spent in the NA were significantly decreased in 5xFAD mice (Figure 8G,H). In the first stage of the new object recognition test, there was no significant difference in sniffing exercise time on the two same things between 5xFAD and WT mice (Figure 8I). However, sniffing exercise time of 5xFAD was significantly decreased in 5xFAD mice compared with WT in the second stage (Figure 8J). Finally, our correlation analysis found that serum iron concentration was negatively correlated with number of astrocytes in the entorhinal cortex and percentage of time spent entering the novelty arm in the Y-maze test (Figure 8K,M), while it was positively correlated with percentage of time spent in the central area (Figure 8L). Meanwhile, number of astrocytes in the entorhinal cortex was negatively correlated with percentage of time spent in the central area (Figure 8N), while it was positively correlated with percentage of time spent entering the novelty arm in the Y-maze test (Figure 8O).

## 3. Discussion

We analyzed more than 13,000 single cells from the entorhinal cortex region of AD patients and healthy controls to demonstrate the landscape of specific cell types in AD and provide knowledge of intercellular communication within the entorhinal cortex region. According to our scRNA-seq analysis, there were significant changes in three cell clusters, including oligodendrocytes, astrocytes, and OPCs. We then performed functional enrichment analysis of DEGs in these three clusters to identify pathways or functions enriched by cell-type-specific changes in AD. Two ferroptosis-related hub mRNAs (FTH1 and SAT1) were consequently identified in astrocytes. Our findings further provided potential interventional targets for AD.

The NRG3–ERBB4 ligand–receptor pair has been reported to be mainly involved in regulating normal cells and tumor cell growth [18]. Apart from that, ablation of NRG3 and ERBB4 led to a reduced excitatory synapse number on parvalbumin-positive interneurons, altered short-term neural plasticity, and disinhibition of the hippocampal network [19]. Here, we suggest that a critical role of the NRG3–ERBB4 ligand–receptor pair was played in intercellular communication in AD. Decreased astrocytes and OPCs resulted in impaired NRG3–ERBB4 ligand–receptor pairs and dysregulation of synapse formation. It would be of great interest to investigate how the NRG3–ERBB4 ligand–receptor pair works in AD on a cell-type-specific basis. Exploring novel brain-penetrant small molecule drugs as NRG substitutes to modulate ERBB receptor signaling would be another perspective in the treatment of AD.

Our results showed that dysregulated pathways in decreased OPCs were related to neuron-to-neuron synapse. Several other studies reported similar findings. Chacon et al. marked OPCs with nuclear dye Hoesch and demonstrated that a decreased number and morphologic changes in OPCs are pathological signs in a mouse model of AD [20]. Vanzulli and colleagues observed quantity and morphologic changes in OPCs at different stages in AD and demonstrated that OPCs disruption is an early pathological sign in AD [21]. We found that oligodendrocytes, myelinating cells in the central nervous system, were enriched in oxidative phosphorylation, which is essential in providing energy for the process of myelination. Therefore, dysfunction of oligodendrocytes may lead to myelination disorders and consequently result in multitype neurodegenerative diseases [22].

**Figure 9:**
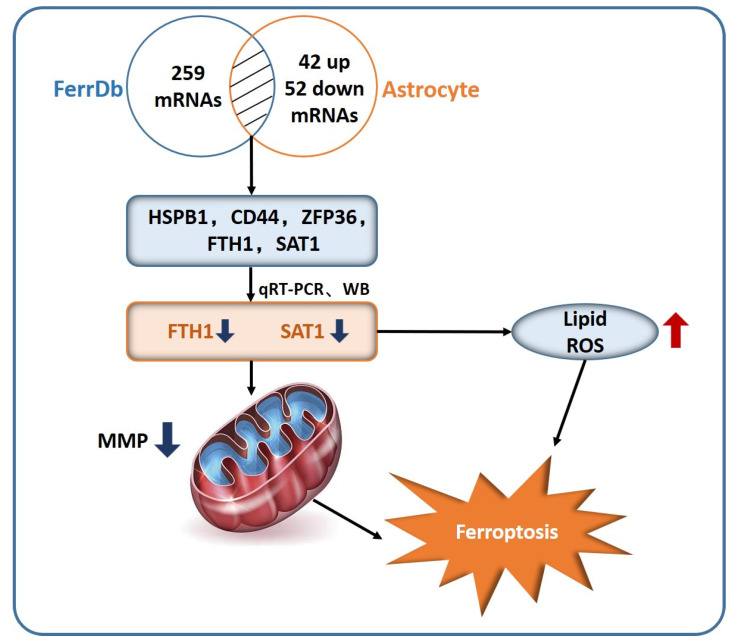
Schematic diagram showing astrocyte ferroptosis in AD. Decreased FTH1 and SAT1 resulted in a decrease in mitochondrial membrane potential and accumulation of lipid ROS and eventually led to astrocyte ferroptosis in AD. MMP: mitochondrial membrane potential.

In addition, we found that dysregulated pathways in astrocytes are related to ferroptosis, which is different from previous single-nucleus RNA-seq studies [17,23,24]. Specifically, we analyzed expression features of the entorhinal cortex region in AD with data from an external dataset. DEGs between AD patients and healthy controls were also central to ferroptosis, which indicated a critical role of ferroptosis played in AD. Distinctive features of ferroptosis, such as iron dysregulation and lipid peroxidation, were reported in AD and other neurodegenerative diseases [25,26,27]. Increased intracellular concentration of iron was reported to enhance β-secretase activity, leading to increased Aβ production [25]. Iron was also reported to bind to Aβ in His6, His13, and His14 amino acid residues, leading to increased neurotoxicity of Aβ [28,29]. Markesbery et al. found increased lipid peroxidation in autopsy samples of AD patients [30], and it resulted in increased 4-HNE levels [31]. However, previous studies at the bulk tissue level seldomly focused on cell-type-specific regulatory complexity. Our scRNA-seq analysis revealed that astrocytes decreased in the entorhinal cortex region, and this can be explained with ferroptosis dysregulation in AD. A recent study reported that NADPH oxidase 4 promoted oxidative-stress-induced lipid peroxidation via impairment in mitochondrial metabolism, leading to ferroptosis of astrocytes [32]. Hence, we interacted DEGs in astrocytes with genes related to ferroptosis in FerrDb. After qRT-PCR and Western blot testing, we detected two potential hub DEGs (FTH1 and SAT1) associated with ferroptosis in astrocytes. FTH1 was responsible for intracellular iron storage and cellular iron metabolism [33]. FTH1 was significantly downregulated in a rat model of Parkinson’s disease compared with controls. Overexpression of FTH1 impaired ferritinophagy (a form of autophagy that degrades ferritin via ferroptosis), ultimately suppressing ferroptosis in PC-12 cells [34]. Activation of SAT1, a rate-limiting enzyme in polyamine catabolism, induced lipid peroxidation and sensitized cells to undergo ferroptosis upon ROS-induced stress, which was found to suppress tumor growth in xenograft tumor models [35]. We then validated the regulation of ferroptosis in AD and found that GPX4 and SLC7A11 were significantly downregulated in the Aβ_25–35_ group. Cell viability, EdU-positive cells, and mitochondrial membrane potential were significantly decreased, while Fe^2+^ and ROS levels increased. Consistently, 5xFAD mice presented higher serum iron levels than WT mice. Behavioral experiments showed that 5xFAD mice had abnormal activity in both the anxiety-like behavior test and short-term cognitive function test. Our correlation analysis showed that serum iron concentration was negatively correlated with number of astrocytes and percentage of time spent entering the NA, while it was positively correlated with percentage of time spent in the central area. Meanwhile, number of astrocytes was negatively correlated with percentage of time spent in the central area, while it was positively correlated with percentage of time spent entering the NA. Our in vitro and in vivo experiments further confirmed the activation of astrocyte ferroptosis in AD (Figure 9), but how these two hub genes (FTH1 and SAT1) affect astrocyte ferroptosis requires further experimental verification.In the current study, we demonstrated that astrocytes underwent ferroptosis in the entorhinal cortex of AD, and the cognitive and behavioral functions of 5xFAD mice also deteriorated compared with WT mice. Serum iron levels were significantly associated with cognitive and behavioral function. This suggests that disease progression in Alzheimer’s patients is closely related to ferroptosis. Meanwhile, we noticed that ferroptosis-related genes (FTH1 and SAT1) were downregulated in astrocytes in the entorhinal cortex of AD. This implicates them as current targets of therapeutic efforts, paving the way for future development of drugs that prevent, delay, or slow progression and target the major pathophysiological mechanisms of AD.

Our study cannot be burdened with limitations. First, we have only depicted the cellular atlas of the entorhinal cortex in AD patients due to the limited open access datasets. In addition, the atlas in the current study represented cells that survived across experimental procedures, so it is assumed that the number of cell clusters in the entorhinal cortex may be underestimated, for example, being lost during cell dissociation and library preparation steps. Although we identified ferroptosis as an essential pathophysiological process in astrocytes, the specific mechanisms underlying ferroptosis in astrocytes need to be further verified with well-designed in vitro and in vivo studies.

## 4. Materials and Methods

### 4.1. Animals

Male 5xFAD mice were kept in a well-ventilated and pathogen-free environment. Mice were maintained under standard laboratory conditions with 40–60% humidity and 20 ± 3 °C temperature [36]. Six 5-month-old 5xFAD mice were assigned to AD group, with 5-month-old wild-type (WT) littermates as controls. All animal procedures followed guidelines and were approved by the Institutional Animal Care and Use Committee of Nanjing Medical University (Reference number 1812054-1) [37].

### 4.2. Cell Culture and Treatment

Normal human astrocytic (NHA) cells were cultured in astrocyte growth medium in an incubator at 37 °C with 95% humid air and 5% CO_2_ according to instruction of Astrocyte Medium Bullet Kit (Lonza, Basel, BS, CH) [38]. For mimic Aβ-induced neuronal injury, cultured cells were treated with Aβ_25–35_ peptide (Aladdin, Shanghai, China) in a concentration of 20 µM for 24 h [39,40].

### 4.3. scRNA & RNA Sequencing Data Processing

“Seurat” package in R software was used for downstream principal component analysis (PCA) and uniform manifold approximation and projection (UMAP) analysis with GSE138852 scRNA sequencing dataset [41]. Gene expression was normalized using “LogNormalize” method. Afterwards, highly variable genes were identified with “vst” method for each sample. PCA was applied to identify significant principal components (PCs), and *p*-value distribution was visualized using “JackStraw” and “ScoreJackStraw” methods. FindClusters function was used to categorize cells into eight different clusters, while FindAllMarkers function with logFC.threshold = 0.25 was applied to identify DEGs for each cluster. Cell type identification was performed based on DEGs in each cluster and was manually checked according to method from a previous study [23]. Volcano plots of DEGs in each cluster were drawn using “GOplot” package [42].

DEGs in GSE5281 RNA sequencing dataset were calculated using “limma” package in R software [43]. DEGs with adjusted *p*-value < 0.05 and absolute logFC > 1 were considered to be significantly dysregulated. Similarly, “GOplot” package was used to produce volcano and heatmap plots.

### 4.4. Cell-to-Cell Ligand–Receptor Interaction Analysis

“CellChat” package was used to predict and visualize biologically significant intercellular communication [44]. Briefly, a CellChat object was made using createCellChat function. After annotating the object with relevant labels and identifying overexpressed genes, computeCommunProb function was used for inferring communication probability. Intercellular communications of each cell signaling pathway were predicted with computeCommunProbPathway function [45,46].

### 4.5. Functional Enrichment Analysis

To further explore potential functions associated with DEGs in oligodendrocytes, astrocytes, and OPCs in GSE138852, or DEGs in GSE5281, GO term and KEGG pathway enrichment were performed using “clusterProfiler” package in R software [47].

### 4.6. Screening Strategy for Ferroptosis-Related Genes in Astrocyte

To explore potential ferroptosis-related genes in astrocytes, we interacted DEGs in astrocytes with genes related to ferroptosis extracted from FerrDb (http://www.zhounan.org/ferrdb/, accessed on 15 May 2022) [48]. Results were visualized by Venn diagram with Venny 2.1.0 (http://bioinfogp.cnb.csic.es/tools/venny/index.html, accessed on 15 May 2022).

### 4.7. qRT-PCR

Total RNA from NHA cells was extracted using Trizol (TaKaRa, Kusatsu, Japan) and cDNA was synthesized using total RNA with PrimeScript™ RT reagent kit (TaKaRa, Kusatsu, Japan) according to manufacturer’s instructions [49]. qRT-PCR was performed with Pro-17 Steponeplus system (Applied Biosystems, Carlsbad, CA, USA). Six pairs of primers are listed in Table S1 (Supplementary File SI). Relative amounts of DEGs were calculated with 2^−^^ΔΔCT^ method [50].

### 4.8. Western Blot

Western blot analysis was performed as previously described [51]. Specifically, NHA cells were lysed using ice-cold lysis buffer (Beyotime, Shanghai, China). Concentration was measured with BCA Protein Assay kit (New Cell and Molecular Biotech, Suzhou, China). After target proteins were loaded onto 10% SDS polyacrylamide gel, they were transferred from gel to PVDF membranes. Subsequently, membranes were blocked with 5% skim milk for 2 h in room temperature and then incubated overnight with GAPDH (Proteintech, Wuhan, China), HSPB1 (Proteintech, Wuhan, China), FTH1 (Immunoway, Suzhou, China), CD44 (Proteintech, Wuhan, China), SAT1 (Proteintech, Wuhan, China), ZFP36 (Proteintech, Wuhan, China), GPX4 (Proteintech, Wuhan, China), SLC7A11 (Proteintech, Wuhan, China), Caspase3 (Proteintech, Wuhan, China), Cleaved-caspase3 (Proteintech, Wuhan, China), Bcl-2 (Abcam, Cambridge, MA, USA), Bax (Proteintech, Wuhan, China), and Cyt-c (Proteintech, Wuhan, China). Afterwards, membranes were incubated with HRP-linked secondary antibody (Abcam, Cambridge, MA, USA) for 1 h. Bands were analyzed with chemiluminescence Western blotting detection system (Tanon, Shanghai, China) [52].

### 4.9. Immunofluorescence Analysis on Cryosections

Immunofluorescence was used to identify astrocytes. Brain sections were washed with cold PBS and fixed in 4% paraformaldehyde for 30 min, then blocked with 5% BSA at 37 °C for 1 h, followed by incubation of anti-glial fibrillary acidic protein (GFAP) antibodies (Abcam, Cambridge, MA, USA) at 4 °C overnight. After washing with PBS, sections were incubated for 1 h with fluorochrome-conjugated secondary antibodies and 4′,6-diamidine-2′-phenylindole dihydrochloride (DAPI, Abcam, Cambridge, MA, USA). They were then washed with PBS and observed using THUNDER Imaging Systems (Leica Microsystems, Wetzlar, Germany) [32].

### 4.10. Cell Counting Kit-8

NHA cells were seeded into 96-well plates (5 × 10^3^ cells/well) in 100 µL of Astrocyte Growth Medium. After conventional culture for 24, 48, or 72 h, 20µL of CCK-8 solution (MedChemExpress, Shanghai, China) was added in each well and kept for an additional 2 h. Absorbance value at 450 nm was measured with enzyme labeling instrument [53].

### 4.11. EdU Assay

EdU was conducted with EdU Proliferation Detection Kit (Ribobio, Guangzhou, China) [54]. Specifically, NHA cells were seeded into 96-well plates with 8 × 10^3^ in each well. After being incubated with 100 μL 50 μmol/L EdU medium for 2 h, cells were fixed in 4% paraformaldehyde for 30 min, followed by 50 μL 2 mg/mL glycine for 5 min. After cultivating with 100 μL of 0.5% Triton X-100, cells were incubated with 100 μL of 1 × Apollo^®^ 567 fluorescent staining solution and 100 μL 1 × Hoechst 33,342 reaction solution in a dark environment. Images were collected from THUNDER Imaging Systems (Leica Microsystems, Wetzlar, Germany).

### 4.12. Determination of ROS Level

Cellular lipid peroxidation was evaluated by using C11-BODIPY581/591 dye. Cells were loaded with 1 mL of 10 μM C11-BODIPY581/591 dye for 30 min at 37 °C. Excitation wavelength of C11-BODIPY581/591 was set at 488 nm (oxidized form) and 563 nm (nonoxidized form) [55,56]. Oxidation of C11-BODIPY581/591 was revealed with change in BODIPY fluorescence from red to green. Fluorescence images were collected from THUNDER Imaging Systems (Leica Microsystems, Wetzlar, Germany).

### 4.13. Fe^2+^ Detection

Level of Fe^2+^ was measured using Mito-FerroGreen kit (DojinDo, Kyushu Island, Japan) [57]. NHA cells were seeded on TC-treated crawlers. Mito-FerroGreen working solution (5 μM, 200 μL) was added and incubated for 30 min. Then, cells were washed with HBSS three times. Finally, cells were observed by THUNDER Imaging Systems (Leica Microsystems, Wetzlar, Germany).

### 4.14. Mitochondrial Membrane Potentials Assay

Mitochondrial membrane potential (MMP) was measured using JC-1 (Beyotime, Shanghai, China) [58]. Specifically, NHA cells were seeded at a density of 1 × 10^4^/well in 6-well plate overnight. Cells were added with 0.5 ml JC-1 working solution (2.5 μg/mL) and incubated for 20 min at 37 °C in dark environment. Then, they were washed with JC-1 staining buffer twice [59,60]. Stained cells were visualized under THUNDER Imaging Systems (Leica Microsystems, Wetzlar, Germany).

### 4.15. Measurement of Iron Indices

Quantitative measurement of serum parameters was performed as previously described [61]. Serum iron concentration was determined using a serum iron assay kit (Catalog A039-1-1, Nanjing Jiancheng Bioengineering institute, Nanjing, China). Total iron binding capacity assay kit (Catalog A039-1-1, Nanjing Jiancheng Bioengineering institute, Nanjing, China) was used to determine total iron binding capacity (TIBC).

### 4.16. Open Field Test

Open field test was performed to evaluate anxiety-like behavior [62]. Apparatus consisted of the open field experiment box (60 × 60 × 25 cm) with an outlined central area (30 × 30 cm). Mice were placed in the middle of the bottom. They were allowed to move freely for 5 min within the box; time spent and distance traveled, as well as number of entrances into the central area, were recorded. After each test, box was cleaned to avoid odors of last mice, which may affect the next animal.

### 4.17. Elevated plus Maze

Elevated maze is a widely used test for measuring anxiety-like behavior [63]. It consists of four intersecting arms (5 × 30 cm), two with walls (“closed”, 15 cm in height) and two without (“open”). The entire apparatus was 40 cm above the ground and was placed in the central area of a room, illuminated from the top (15 lx). Mice were placed in the center of the maze and allowed to freely explore for 5 min. Video clips were captured and analyzed for time spent in open and closed arms, as well as total number of arm entries. An animal was considered in an arm whenever the body (not including the tail) was completely in the arm. Maze was thoroughly cleaned with 70% ethanol after each trial [64].

### 4.18. Y-Maze Test

Y-maze test was conducted to evaluate short-term working memory [65]. Testing occurred in a Y-shaped maze with three arms, including the novel arm (NA), the starting arm (SA), and the other arm (OA). This test contains two 5 min stages with an interval of 2 h between evaluation periods. During the first stage, the NA was blocked by a black baffle, and mice were placed in the starting arm to explore freely for 5 min. At the second stage, the NA was open and the mice could move freely throughout three arms for 5 min. Percentage of time traveled in each arm and number of entries into each arm were recorded.

### 4.19. Novel Object Recognition Test

Novel object recognition test is a widely used behavioral task for investigation of learning and memory in mice [66]. A camera placed above the open field recorded the movements of mice throughout the trial. To test recognition, mice were habituated in an empty box (40 × 50 × 50 cm) and were given 5 min to explore two identical objects in the recognition box. After 1 h, one of the objects was replaced with a novel object and mice were again placed in the maze for free exploration for 5 min. The discriminant index was calculated using the following formula: (time spent on the new object/total time spent on the two detection objects) × 100%. Exploration was defined as when mouse’s nose pointed towards the object within 2 cm.

### 4.20. Statistical Analysis

Data were presented with mean and standard deviation. Statistical significance was considered with *p* value less than 0.05. T-test was used for results of qRT-PCR, iron indices, TIBC, open field test, Y-maze test, novel object recognition test, and elevated plus maze. Two-way ANOVA was used in the CCK-8 experiments. Statistical analyses were performed with GraphPad Prism 9 (GraphPad, San Diego, CA, USA) and SPSS 22.0 (International Business Machines Corporation, Armonk, NY, USA).

## 5. Conclusions

With the assistance of the advanced scRNA-seq technique, we revealed cellular heterogeneity and highlighted ferroptosis as an essential pathophysiological process in astrocytes in the entorhinal cortex region of AD. Two hub mRNAs (FTH1 and SAT1) were confirmed to affect the process of ferroptosis in astrocytes. Emotional and cognitive impairment in AD was associated with astrocyte ferroptosis. Our findings provided novel perspectives in revealing the pathophysiological process after AD at the cellular level and highlighted potential drug targets for treatment of AD.

## Data Availability

Data is contained within the article and Supplementary Material.

## Supplementary Materials

The following supporting information can be downloaded at: https://www.mdpi.com/article/10.3390/ph15101177/s1, Table S1 in Supplementary File SI. Primer sequences of RNAs for qRT-PCR. Table S2 in Supplementary File SI. Cell numbers of each cluster. Supplementary File SⅡ. Genes list of eight clusters. Supplementary File SIII. Results of cell–cell communication analysis in HC. Supplementary File SⅣ. Results of cell–cell communication analysis in AD.
