# Supplementary material for: FTH1- and SAT1-Induced Astrocytic Ferroptosis Is Involved in Alzheimer’s Disease: Evidence from Single-Cell Transcriptomic Analysis
Source: Pharmaceuticals (Basel). 2022 Sep 22;15(10):1177. doi: 10.3390/ph15101177 (PMC9610574; doi:10.3390/ph15101177)
Supplement: Supplementary file 1 [file pharmaceuticals-15-01177-s001.zip › Supplementary File Só±.pdf]

Supplementary File I

List of content

Table S1. Primer sequences of RNAs for qRT-PCR
Table S2. Cell numbers of each cluster

Table S1. Primer sequences of RNAs for qRT-PCR

Gene Name	Species	Sequence
GAPDH	Human	F:5' CTGGGCTACACTGAGCACC 3'
		R:5' AAGTGGTCGTTGAGGGCAATG 3'
HSPB1	Human	F:5' CAGGACGAGCATGGCTACAT 3'
		R:5' TGATCTCGTTGGACTGCGTG 3'
FTH1	Human	F:5' CCCCCATTTGTGTGACTTCAT 3'
		R:5' GCCCGAGGCTTAGCTTTCATT 3'
CD44	Human	F:5' CACACCCTCCCCTCATTCAC 3'
		R:5' TGGATGGCTGGTATGAGCTG 3'
SAT1	Human	F:5' CCTGACTGAGAAGAGGACGC 3'
		R:5' TGATCAGCCGCAGTATGTCA 3'
ZFP36	Human	F:5' ACTGCCATCTACGAGAGCCT 3'
		R:5' ACTAGGCTGGTGGAGCGG 3'

Table S2. Cell numbers of each cluster

cluster	Oligo	Astro	OPC	Neuron	UnID	Mg	Doublet	Endo
AD1-AD2	1956	96	32	99	540	21	279	5
AD3-AD4	2075	235	57	114	26	87	9	2
AD5-AD6	624	141	90	36	48	64	7	30
HC1-HC2	409	166	88	68	279	54	47	11
HC3-HC4	1431	853	427	124	10	142	24	32
HC5-HC6	937	680	384	215	22	81	39	18

HC: healthy controls. AD: Alzheimer's disease. Astro: astrocytes. UnID: unidentified cells. Oligo: oligodendrocytes. Endo: endothelial cells. Mg: microglia.
